# Prediction and Validation of Transcription Factors Modulating the Expression of Sestrin3 Gene Using an Integrated Computational and Experimental Approach

**DOI:** 10.1371/journal.pone.0160228

**Published:** 2016-07-28

**Authors:** Rajneesh Srivastava, Yang Zhang, Xiwen Xiong, Xiaoning Zhang, Xiaoyan Pan, X. Charlie Dong, Suthat Liangpunsakul, Sarath Chandra Janga

**Affiliations:** 1 Department of Biohealth Informatics, School of Informatics and Computing, Indiana University Purdue University, 719 Indiana Ave Ste 319, Walker Plaza Building, Indianapolis, Indiana, 46202, United States of America; 2 Department of Biochemistry and Molecular Biology, 635 Barnhill Drive, Indianapolis, Indiana, 46202, United States of America; 3 Department of Clinical Laboratory, Shandong Provincial Qianfoshan Hospital, 16766 Jingshi Road, Jinan, Shandong Province, 250014, China; 4 Division of Endocrinology, The First Affiliated Hospital of Wenzhou Medical University, Wenzhou, Zhejiang Province, 325015, China; 5 Division of Gastroenterology and Hepatology, Department of Medicine, Indiana University, Indianapolis, Indiana, 46202, United States of America; 6 Roudebush Veterans Affairs Administration Hospital, Indianapolis, Indiana, 46202, United States of America; 7 Center for Computational Biology and Bioinformatics, Indiana University School of Medicine, 5021 Health Information and Translational Sciences (HITS), 410 West 10th Street, Indianapolis, Indiana, 46202, United States of America; 8 Department of Medical and Molecular Genetics, Indiana University School of Medicine, Medical Research and Library Building, 975 West Walnut Street, Indianapolis, Indiana, 46202, United States of America; The University of North Carolina at Charlotte, UNITED STATES

## Abstract

*SESN3* has been implicated in multiple biological processes including protection against oxidative stress, regulation of glucose and lipid metabolism. However, little is known about the factors and mechanisms controlling its gene expression at the transcriptional level. We performed in silico phylogenetic footprinting analysis of 5 kb upstream regions of a diverse set of human *SESN3* orthologs for the identification of high confidence conserved binding motifs (BMo). We further analyzed the predicted BMo by a motif comparison tool to identify the TFs likely to bind these discovered motifs. Predicted TFs were then integrated with experimentally known protein-protein interactions and experimentally validated to delineate the important transcriptional regulators of *SESN3*. Our study revealed high confidence set of BMos (integrated with DNase I hypersensitivity sites) in the upstream regulatory regions of *SESN3* that could be bound by transcription factors from multiple families including *FOXOs*, *SMADs*, *SOXs*, *TCFs* and *HNF4A*. TF-TF network analysis established hubs of interaction that include *SMAD3*, *TCF3*, *SMAD2*, *HDAC2*, *SOX2*, *TAL1* and *TCF12* as well as the likely protein complexes formed between them. We show using ChIP-PCR as well as over-expression and knock out studies that *FOXO3* and *SOX2* transcriptionally regulate the expression of SESN3 gene. Our findings provide an important roadmap to further our understanding on the regulation of *SESN3*.

## Introduction

Sestrins belong to a small family of evolutionally conserved proteins. They are distinct from any other characterized eukaryotic protein families because they do not have any previously identified domain structures[1]. Mammals express three sestrin genes (*SESN*1/2/3), while most invertebrates contain only a single sestrin gene[2]. Sestrins do not contain any known structural domains/catalytic motifs; only a partial homologous sequence to bacterial oxidoreductases is identified, suggesting an antioxidant function of this protein[1]. Sestrins regulate multiple signaling pathways for metabolic and cellular homeostasis[3]. First, sestrins reduce oxidative stress through either their intrinsic oxidoreductase activity or *NRF2* (nuclear factor erythroid derived 2 like 2)-regulated pathway [4,5]. Second, sestrins modulate glucose and lipid metabolism through *AMPK* (AMP-activated protein kinase) and *mTORC1* (mechanistic target of rapamycin complex 1)[1]. Third, Sestrins regulate autophagy through activation of AMPK and inhibition of *mTORC1* [2]. Deletion of a single *SESN* gene in fruit fly leads to triglyceride accumulation in its body [2], equivalent to the liver in mammals. We have observed that ethanol suppresses *SESN3* gene expression and function in hepatocytes and mouse livers. Over expression of *SESN3* dramatically reduces the ethanol-induced hepatic steatosis [6]. In addition, *SESN2* and *SESN3* have also been shown to regulate insulin sensitivity and glucose homeostasis [7,8]. However, to date, the factors that control *SESN3* expression are not well studied. Understanding the complex regulatory mechanisms that regulate the *SESN3* is of importance, as new therapeutic targets for metabolic diseases might be discovered.

Transcription factors (TFs) are known to bind specifically to gene’s promoters at the regulatory positions (binding motifs) and thus contribute to its transcriptional regulation and cellular function. Various *in vitro* [9], *in vivo* [10] and *in silico* [11] approaches have been developed for identifying TF binding motifs. Typically, potential TFs bind to its high affinity binding sites (represented as a weight matrix), however, little is known about the tissue specific binding pattern of most TFs in higher eukaryotes [12].

In this study, we used the upstream regulatory regions of human *SESN3* orthologs from a diverse set of primates and rodents (with at least 85% sequence homology with human) to perform phylogenetic footprinting [13]. We employed the MEME-SUITE of tools [14,15] which allowed the identification of high confidence conserved binding motifs and corresponding position specific weight matrices. We also tested the feasibility (i.e. TF binding tendency) of these binding motifs (BMo) in open chromatin region of human cell lines and mouse liver using DNase Hypersensitive Sites (DHS) in *SESN3* upstream region. Predicted binding motifs were further analyzed by Tomtom (a motif comparison tool from MEME-SUITE) to identify motif specific potential transcription factors. Predicted TFs were integrated with documented protein-protein interaction in BioGRID [16] to decipher the important regulators and the network of interactors controlling the expression of the *SESN3* gene.

## Materials and Methods

Human-*SESN3* orthologs and their upstream regulatory regions were extracted (FASTA sequences) from ENSEMBL. These *SESN3* sequences from human and its 10 orthologs (Primates and Rodents) were taken and executed using MEME-SUITE, an open source hub of bioinformatics tools. Prediction of novel regulatory motifs was performed by using phylogenetic footprinting, an in silico method coupled with downstream computational analysis. Based on this, consensus sequences in upstream region were discovered by MEME analysis. These consensus sequences were further analyzed using the Tomtom tool which enables the comparison of predicted motifs with Position Weight Matrices (PWM) of TFs for overlap. Further, protein-protein interaction network was constructed between the potential TFs by utilizing the available physical interactions in BioGRID to delineate the important regulators and the network of interactors controlling the expression of *SESN3* gene.

### Sestrin 3 transcripts and their expression profile

Human *SESN3* gene is located on chromosome 11. We obtained DNA sequences for the human *SESN3* gene (Ensembl ID ENSG00000149212) from the ENSEMBL database. There are 5 transcripts reported for the human *SESN3* gene (Table 1); of which 4 have been reported to be protein coding. Expression profile of this gene was obtained from open source database–GeneCards [17], for further examination.

**Table 1:** Transcripts of Human *SESN3* gene reported in ENSEMBL database.

Name	Transcript ID	Length	Protein	Biotype
***SESN3*-001**	ENST00000536441	9531 bp	492 aa (view)	Protein coding
***SESN3*-003**	ENST00000278499	1710 bp	353 aa (view)	Protein coding
***SESN3*-002**	ENST00000416495	1408 bp	321 aa (view)	Protein coding
***SESN3*-004**	ENST00000542176	607 bp	70 aa (view)	Protein coding
***SESN3*-005**	ENST00000537480	541 bp	No protein product	Processed transcript

### Identification of human *SESN3* orthologs and their upstream regulatory regions for phylogenetic footprinting

Phylogenetic footprinting is one of the classical methods applied for DNA binding motif discovery [13,18,19]. It involves the upstream regulatory sequence of a gene of interest across possible orthologs to search for highly conserved consensus DNA binding sites. We selected orthologs of the human *SESN3* gene from primates and rodents using Ensembl Compara gene trees [20]. These dataset allows the identification of orthologous sequences across species with high sequence resemblance as shown in S1 Table. Gene expression is controlled by various cis-acting transcriptional regulatory factors by binding mostly in close proximity to the transcription start sites (TSS) in the promoter regions of a gene [21]. Based on previous studies from others [22,23] and our group, we found that most functional TF binding sites occur within the 5kb upstream region of the gene TSS (data not shown). So we focused our study on *5kb upstream regions* of the *SESN3* gene for motif discovery. Upstream regulatory regions for human and its 10 selected *SESN3* orthologs were obtained from Ensembl database (S1 Table).

### MEME analysis for discovering DNA binding motifs

DNA binding motif discovery using the *in silico* phylogenetic footprinting approach covered regulatory regions in the promoters of orthologous genes from multiple species. This is under the notion that regulatory elements would be conserved in the background of non-functional sequences and hence could be discriminated as footprints contributing to regulatory control. To facilitate the motif finding in these regions, we used the MEME-suite of tools [14, 15]. MEME is a tool for discovering motifs in a group of related DNA or protein sequences, which detects the frequently occurring conserved sequence across a group of related DNA sequences, using expectation maximization[24]. These motifs are typically represented as position-dependent letter-probability matrices in logos which describe the probability of each possible letter at each position in the pattern to incorporate the variation in the detected motif instances across sequences. In this study, we used 5kb upstream sequences of human *SESN3* and its 11 orthologs compiled as a FASTA file and used as an input data for MEME to identify significantly over-represented motifs (E-value < e^-34^). Here we limit the width of discovered binding motifs in MEME analysis to reflect the widths of most established PWMs—which typically vary in length between 4bp to 30bp [25–28].

### Prediction of TFs associated with discovered motifs

Transcription Factors (TFs) are thought to bind specifically to their corresponding binding motif (BMo)[18] and regulate the expression of a target gene. DNA binding motifs were represented as PWM (Position-Specific Weight Matrix) based logos. Nucleotide constituent of each consensus motif has its own probability of occurrence within the site. Since PWMs for various TFs have already been reported in JASPAR [25], UniPROBE [26], Jolma et al [27] and TRANSFAC [28] public databases, based on a comparison of the similarity between the reported PWM of a TF to the footprinted PWM in the orthologous upstream regions, it is possible to predict the TFs which are most likely to bind to these predicted binding sites. Tomtom [29] is a tool in the MEME-suite which compares discovered DNA motifs to known motifs of such databases.

We used a set of 2201 DNA motifs ranging between 4bp and 30bp in length (average length 12.7) from TRANSFAC, 843 DNA motifs ranging between 7bp and 23bp in length (average length 12.7) in Jolma et al and 979 DNA motifs ranging between 5bp and 30bp in length (average length 13.0) in JASPAR CORE and UniPROBE Mouse. Hence, we rationalized that a motif length between 4bp to 30bp for the discovered motifs, would be able to capture most of these recognition sequences in the *SESN3* upstream regions.

PWMs of various discovered motifs were used as input file for Tomtom and compared with already reported PWMs of TFs in the above described databases to identify the potential TFs binding to the *SESN3* upstream regions. Only the TF associations which are identified at p ≤ 1e^-03^ with E-value < 10 were considered as statistically significant for the 5kb upstream regions.

### Analysis of DNase I hypersensitive site in *SESN3* upstream region

DNase I hypersensitive sites (DHS) are open chromatin region of DNA, sensitive to DNase I cleavage. It is believed that, the occurrence of DHS, notably in the promoter region [30] is an indicator of potential binding site for transcription factor. We extracted the available DHS data in various human cell lines and mouse (14.5 days and 8 week) liver from ENCODE project [31] and visualized them for upstream regions of *SESN3* genes in UCSC genome browser (http://genome.ucsc.edu/cgi-bin/hgFileUi?db=mm9&g=wgEncodeUwDnase). The images generated from the browser were positioned according to the coordinate of the *SESN3* upstream region of block diagram and studied for active BMo.

### Experimental validation of potential transcription factors

Human HEK293 cells were transfected with plasmid DNAs carrying coding sequences for control GFP (green fluorescent protein), human *FOXO3* and *SOX2* genes. The constructs also contained FLAG tag sequence on the N-terminus. After 48 hours of transfection, cells were processed for chromatin immunoprecipitation (ChIP) analysis for the predicted TF binding sequences as previously described [32]. The sequences for the PCR primers are: *FOXO3* ChIP forward primer 5’-ACAAATCCTGGTACGCTGGA-3’, reverse primer 5’–CAGGACTGTGCATTATGACATCA– 3’; *SOX2* ChIP forward primer 5’–CCAGTAGGCGATGCAAGTTA– 3’, and reverse primer 5’–CTAGACGCCCGCAACCTG– 3’.

### CRISPR/*Cas9* gene knockout

Human *FOXO3* and *SOX2* CRISPR/Cas9 single guide RNA (sgRNA) sequences were designed using an online program at crispr.mit.edu (Dr. Feng Zhang lab) for gene knockout. The selected two sgRNA sequences for the human *FOXO3* and *SOX2* genes are: 5’-CACTTCGAGCGGAGAGAGCG-3’ (*FOXO3* sgRNA1), 5’-TCCACTTCGAGCGGAGAGAG-3’ (*FOXO3* sgRNA2), 5’-TGGGCCGCTTGACGCGGTCC-3’ (*SOX2* sgRNA1), and 5’-ATGGGCCGCTTGACGCGGTC-3’ (*SOX2* sgRNA2). The DNA oligonucleotides were cloned into a lentiCRISPRv2 vector (a gift from Dr. Feng Zhang, Addgene plasmid #52961) as described previously [33,34]. To generate gene knockout stable cell lines, we transfected HEK293T cells with control *GFP*, *FOXO3*, or *SOX2* sgRNA plasmids. The transfected cells were selected using puromycin (1 μg/ml) for 7 days, and then maintained in the culture medium containing 0.5 μg/ml puromycin.

### DNA constructs preparation

The coding sequences for *GFP*, human *FOXO3*, and *SOX2* genes were cloned into a pcDNA3 vector using PCR amplification and restriction digestion.

### Cell culture and transfection

Human HEK293T and HepG2 cells were cultured in DMEM/high glucose medium containing 10% FBS. HEK293T cells were transfected with plasmid DNA using polyethylenimine and HepG2 cells were transfected using TurboFect reagent (Thermo Fisher Scientific).

### mRNA analysis

Total RNAs were isolated from cultured cells using TRI Reagent (Sigma). mRNA levels for selected genes were analyzed by real-time PCR. Peptidylprolyl isomerase A (*PPIA*) was chosen as an internal control gene. Primer sequences are listed as follows: human *PPIA* forward primer: 5’- AGGTCCCAAAGACAGCAGAA-3’, human *PPIA* reverse primer: 5’-GAAGTCACCACCCTGACACA-3’, human *SESN3* forward primer: 5’-GTACCAACTGCCGGAAAGTG-3’, and human *SESN3* reverse primer: 5’- CCACTGTGTTTGCTTGGACA.

### Mapping protein interactions between the potential TFs

Eukaryotic TFs often regulate the expression of genes by forming protein complexes and several examples have been documented in the literature including that of *FOXOs* interacting with *SMAD3* [35], *HNF4a*[36] etc to modulate the transcription of their target genes. We employed the currently available manually curated set of protein-protein interactions for the human genome available from the BioGRID database [37] to map the physical associations between the predicted TFs from the Tomtom analysis for the 5kb upstream region. This not only allowed the construction of a protein interaction network between the predicted TFs but allowed the dissection of the major TFs based on their number of protein interactions in the network.

## Results and Discussion

*SESN3* has similar pattern of expression (RNA seq based) across most of the body fluids like blood, liver secretome, and multiple tissue types (S1 Fig, GeneCards[17]) indicating the consistent and universal transcriptional regulation of this gene. However, little is known about the factors and mechanisms controlling its expression. Our study attempts to identify the cis-regulatory binding sites controlling *SESN3* and all possible regulatory proteins which may be involved in regulating the expression of *SESN3* gene at transcriptional level.

### Identification of potential binding motifs by in silico phylogenetic footprinting in the regulatory regions of *SESN3* across primates and rodents

**Fig 1:**
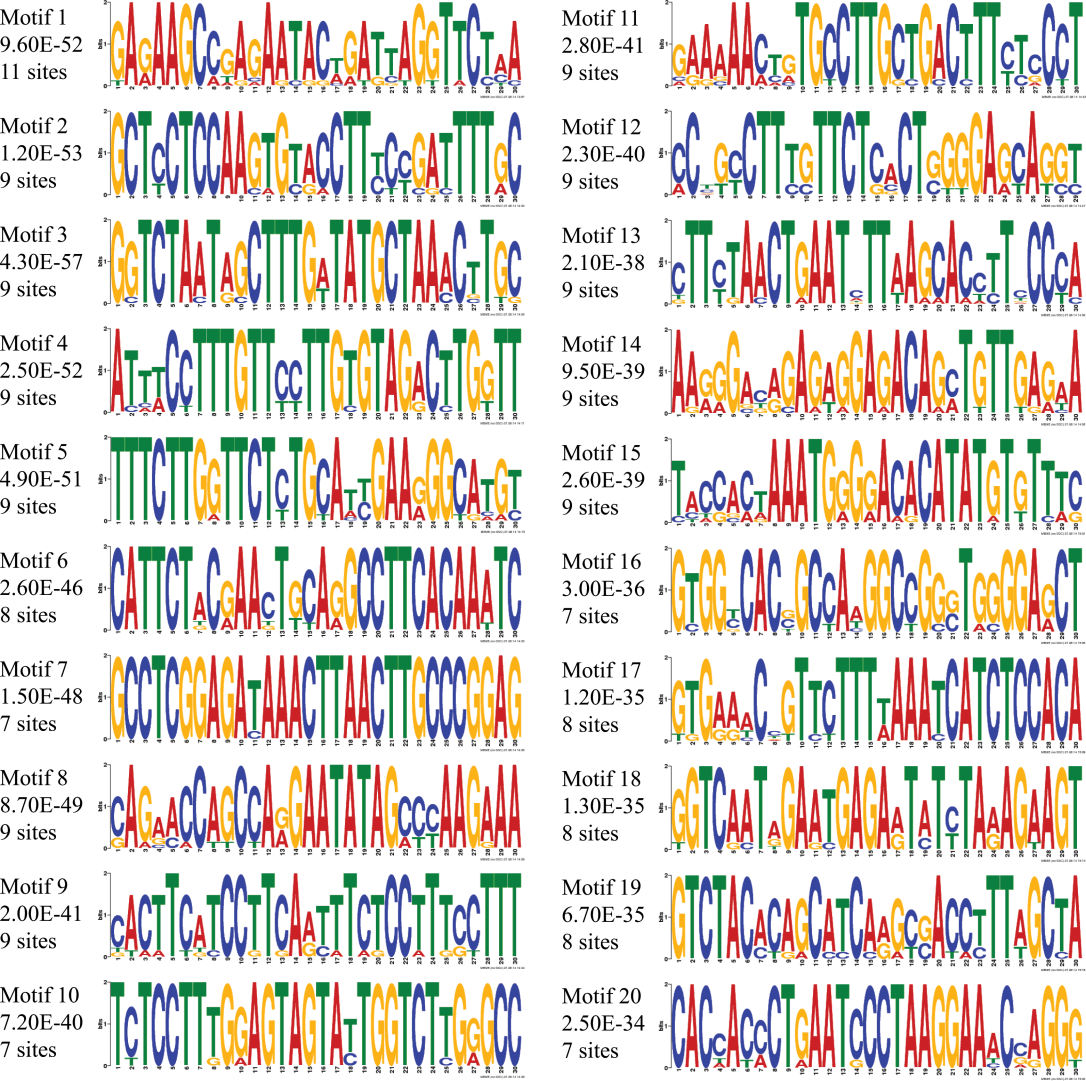
Identification of potential binding motifs by phylogenetic footprinting of 5 kb upstream regulatory regions of *SESN3* gene. Twenty phylogenetically conserved and statistically significant (indicated by e-value) novel motifs with the number of sites contributing to their identification were shown for *SESN3* 5kb upstream. These motifs were displayed as sequence LOGOs representing position weight matrices of each possible letter code occuring at particular position of motif and its height representing the probability of the letter at that position multiplied by the total information content of the stack in bits.

Human *SESN3* consists of multiple protein coding transcripts as outlined in Table 1 extracted from ENSEMBL database. In Ensembl, a gene start refers to the earliest start co-ordinate of all the transcripts associated with a given gene. Phylogenetic footprinting analysis facilitates the search for regions of conserved chromosomal fragments where the likelihood of transcription factor binding is high. These protein-binding sites, which are short fragments of DNA, often range from 6–30 bp in length[18, 38–40]. We identified the set of binding sites and corresponding TFs controlling the *SESN3* gene by performing motif discovery based on phylogenetic alignments of orthologous sequences from a diverse set of primates and rodents using the human *SESN3* gene as a reference (see Materials and Methods, S1 Table). *In silico* phylogenetic footprinting [18], was applied for identifying the best conserved motifs in those orthologous regions [13]. This approach has its shortcoming as it may miss some of the binding motifs which are not conserved in upstream region of *SESN3*. However, this approach has several advantages because of the limited information currently available regarding the transcription regulators controlling this gene. Further, this analysis also limits the false discovery of motifs as well as associated TFs. Briefly, 5kb upstream sequences of *SESN3* gene for human and its orthologs (S1 Table) were analyzed by MEME, an expectation maximization-based motif-finding algorithm, to identify the potential binding sites conserved across the species. We have used the gene start as the reference to obtain the 5kb upstream. Based on the alignments, PWMs representing each of the 20 most significant BMo enriched across the analyzed sequences were identified. We observed that most of the established binding motif PWMs in publically available databases ranges in length between 4 bp to 30 bp (See Materials and Methods) therefore, we believe that the discovered motifs in current study would be able to capture most of these recognition sequences, including large co-complex TF binding sites or palindrome motifs, if they are present in the *SESN3* upstream. Motif logos[41] corresponding to each of these 20 significantly conserved ones along with the number of occurrences of the motifs across the 11 sequences were shown in Fig 1. Consensus sequences representing these discovered motifs were shown in S2 Table.

### Distribution of binding motifs for *SESN3* across species

**Fig 2:**
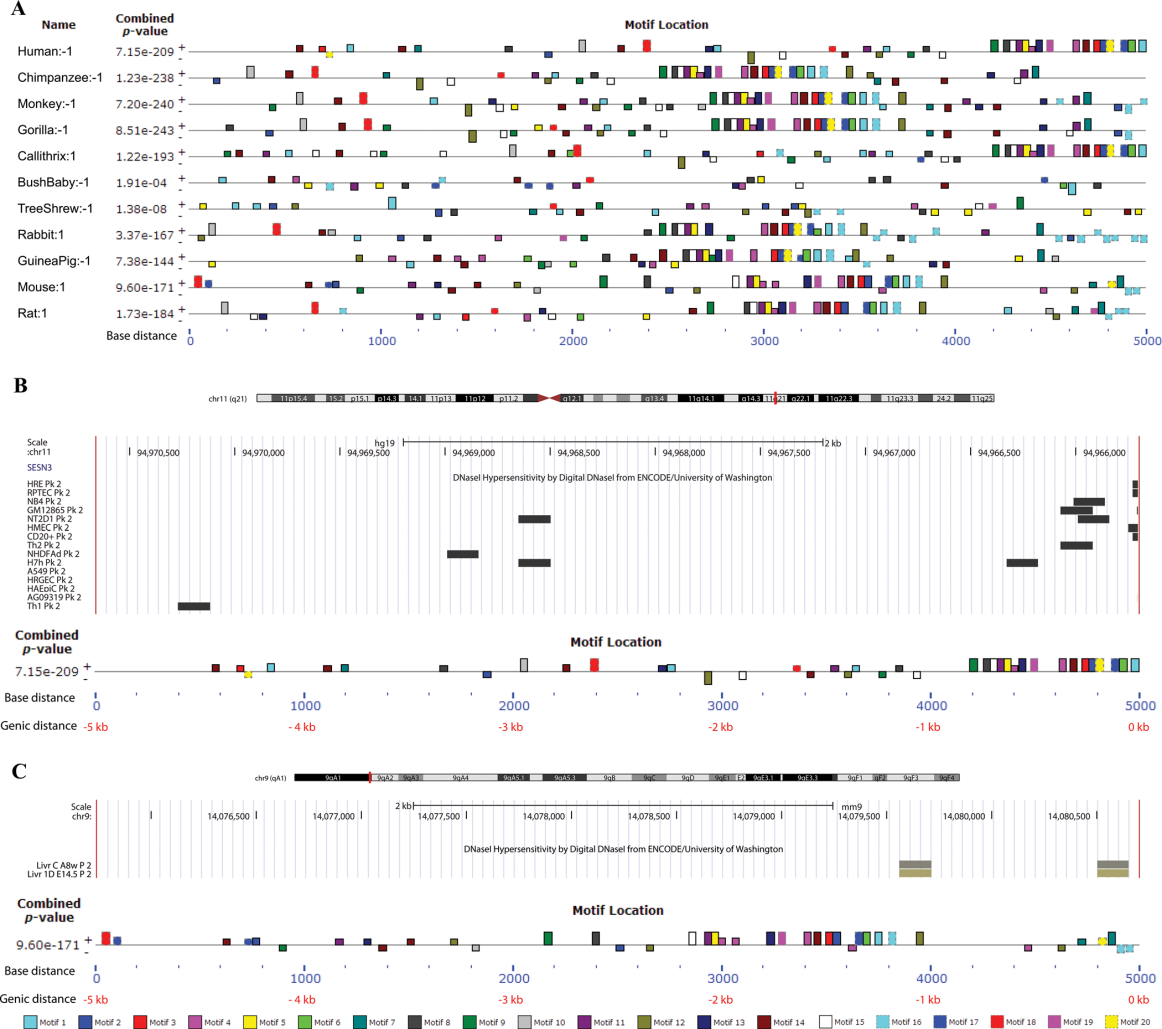
Block diagram showing occurrence of conserved motifs. **(A)** Location of twenty motifs identified and their distribution in 5 kb upstream sequences across human-*SESN3* & its other primate/rodent orthologous species were shown in the block diagram. The combined best matches of a sequence to a group of motifs were shown by combined p value. Sequence strand specified as “+” (input sequence was read from left to right) and “-” (input sequence was read on its complementary strand from right to left) with respect to the occurrence of motifs. Coordinates of each motif across species is shown as a sequence scale (from left to right, in blue) below the diagram. DNase I hypersensitive region was shown in 5kb upstream region of *SESN3* in **(B)** human cell lines and **(C)** mouse liver (8 week adult and 14.5 days embryo) using ENCODE project, represented by UCSC browser visualization tool. An overlap of DHS signal was found and shown as dark band over respective motifs in block diagram. The two coordinates on x-axis represents the *5kb upstream regions* as base distance (in blue) and genic distance (with respect to gene start site, in red) of *SESN3* gene.

Genes of many eukaryotes display a more complex architecture of associated regulatory elements, including cis-promoter elements with binding sites for basal transcription factors, and distal /trans elements with host specific transcription factors binding sites [42]. Several elegant studies on developmentally regulated [43] and immune-response genes [44,45] have revealed an important role for combinatorial interactions between different transcription factors (TFs) in establishing the complex sequential patterns of gene expression. Hence, increasing evidence now suggests the importance of not only knowing the binding location of a eukaryotic TF [46] but also the complex combinatorial interplay between them [47]. Therefore, we first mapped the identified conserved novel motif sites across multiple species. These binding motifs were quite different from each other; as indicated by the Pearson correlation coefficient values (S3 Table) obtained using MAST from MEME-suite[15, 48]. Relative positions of the discovered binding sites in the 5kb upstream regulatory sequences across the species, organized by phylogenetic distance along with the combined significance of motif co-occurrence, were shown as a block diagram (Fig 2A). The conservation of motifs was observed high in the region between -1 and -2.5 kb of the *SESN3* gene promoter.

DNase I hypersensitive sites (DHSs) are DNase I enzyme sensitive regions of chromatin, where chromatin has less condensed structure due to chromatin remodeling for facilitating transcriptional activation and other downstream events [49]. We used the DHS data available for human cell lines and mouse liver (14.5 days and 8 weeks), generated from University of Washington as part of the ENCODE project [50]. Our analysis strongly suggested several predicted motifs (Fig 2B and 2C) in 5 kb upstream region of the *SESN3* genes to be active and open for transcription factor binding, especially within 1 kb of the gene promoter.

### Prediction and validation of transcriptional regulatory apparatus targeting discovered motifs of *SESN3* upstream region

**Fig 3:**
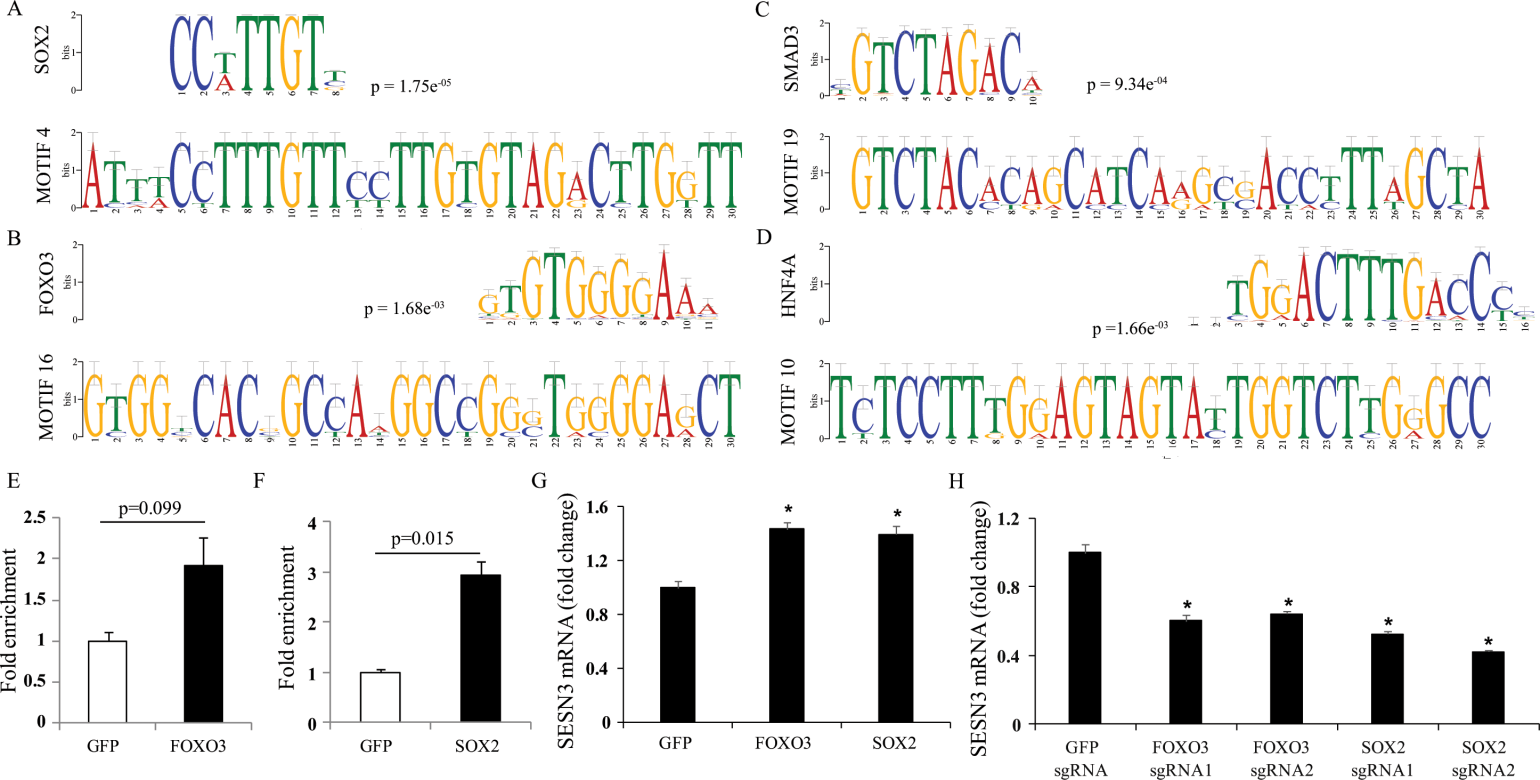
Tomtom analysis results for conserved motifs and experimental validation. **(A-D)** Transcription factors predicted for 20 consensus sequences (as query motif) by Tomtom analysis. Selected set of DHS overlapped motif aligning with their TF’s PWM (top) and query motif (bottom) with binding specificity mentioned by p-values. **(E-F)** Validation of *FOXO3* and *SOX2* binding to predicted BMo location in *SESN3* upstream region by ChIP analysis. **(G)** Overexpression of *FOXO3* and *SOX2* activated the *SESN3* gene expression in human HepG2 hepatoma cells. **(H)** Knockout of *FOXO3* or *SOX2* using CRISPR/Cas9 approach downregulated the *SESN3* gene in human HEK293 cells. (* p<0.05).

We downloaded the motif databases viz. JASPAR CORE 2014, TRANSFAC, UniPROBE mouse and Jolma 2013 (See Materials and Methods) separately and then combined all together to perform the motif comparison analysis using Tomtom with proper filtering criteria (p-value ≤ 1e^-03^ and E-value <10). All possible TFs predicted to bind to the discovered motifs were catalogued and shown in S4 Table. High confidence set of TFs predicted to regulate the expression of SESN3 via Tomtom [29] included *FOXOs*, *SMADs*, *SOXs*, *HNF4A*, and *TCFs* (see S4 Table, Fig 3A–3D). We validated binding motifs which corresponded to high confidence TFs overlapping with DHS signals viz. *SOX2* and *FOXO3* using ChIP-PCR approach in HEK293 cells (See Materials and Methods). *SOX2* and *FOXO3* transcription factors were found to exhibit significantly enriched binding to the predicted location in the human *SESN3* promoter region compared to a negative control *GFP* (Green Fluorescent Protein) (Fig 3E and 3F). Thus, this validation confirms the active BMos discovered for *FOXO3* and *SOX2* in the promoter region of the human *SESN3* gene. To further verify the functional relevance of these TFs in the regulation of the *SESN3* gene, we also performed overexpression and knockout of *FOXO3* and *SOX2* in human cell lines. We found that overexpression of *FOXO3* or *SOX2* could significantly activate the *SESN3* gene in human HepG2 hepatoma cells (Fig 3G), but they did not significantly affect the *SESN3* gene expression in human HEK293 cells (embryonic kidney cell line) (S2 Fig), suggesting that there might be cell-type-specific effects. Nevertheless, knockout of either *FOXO3* or *SOX2* downregulated the *SESN3* gene expression (Fig 3H).

There are different isoforms of *SESN3* as shown in Table 1. Therefore, it is possible to have alternative regulatory elements in the first intron of the gene. In addition to the previous analysis, we also performed insilico phylogenetic foot printing with 3 kb upstream and 2 kb instream query sequence of the primates and rodents for motif discovery and potential TFs binding to these new motifs. The new analysis might not produce the same set of motifs similar to the previously identified consensus sequences because the sequence search spaces are different, however we believe, motifs which overlap fully or partially with common DHS signals to the previous analysis, should produce reproducible results. We identified a set of 20 overrepresented consensus motifs (E-value < e^-44^) among which, motifs overlapping with the DHS signals (See S3 and S4 Figs), and their corresponding potential binding transcription factors are documented in S5 Table. We observed that ~64% of the previously detected TFs (whose binding motifs were supported with DHS) were still detected in the new analysis including *SOXs*, *FOXOs*, *SMADs*, *TCFs*, *HAP1*, *LEF1*, *GATA1*, *POU3F4*, *POU5F1*, *EKLF* and *TFAP4*. Hence, inclusion of instream region increased the coverage of predicted TFs in our analysis corresponding to the newly identified motifs. Additionally, we examined the length distribution of TF sites overlapping with the width of discovered motifs. We observed that most TF binding sites exhibited an overlap between 4–28 bp with the query motifs as shown in S5 Fig, suggesting that employing a 6–30 bp motif width is an ideal threshold to capture most potential TFs likely to bind to the upstream regions of *SESN3*.

**Fig 4:**
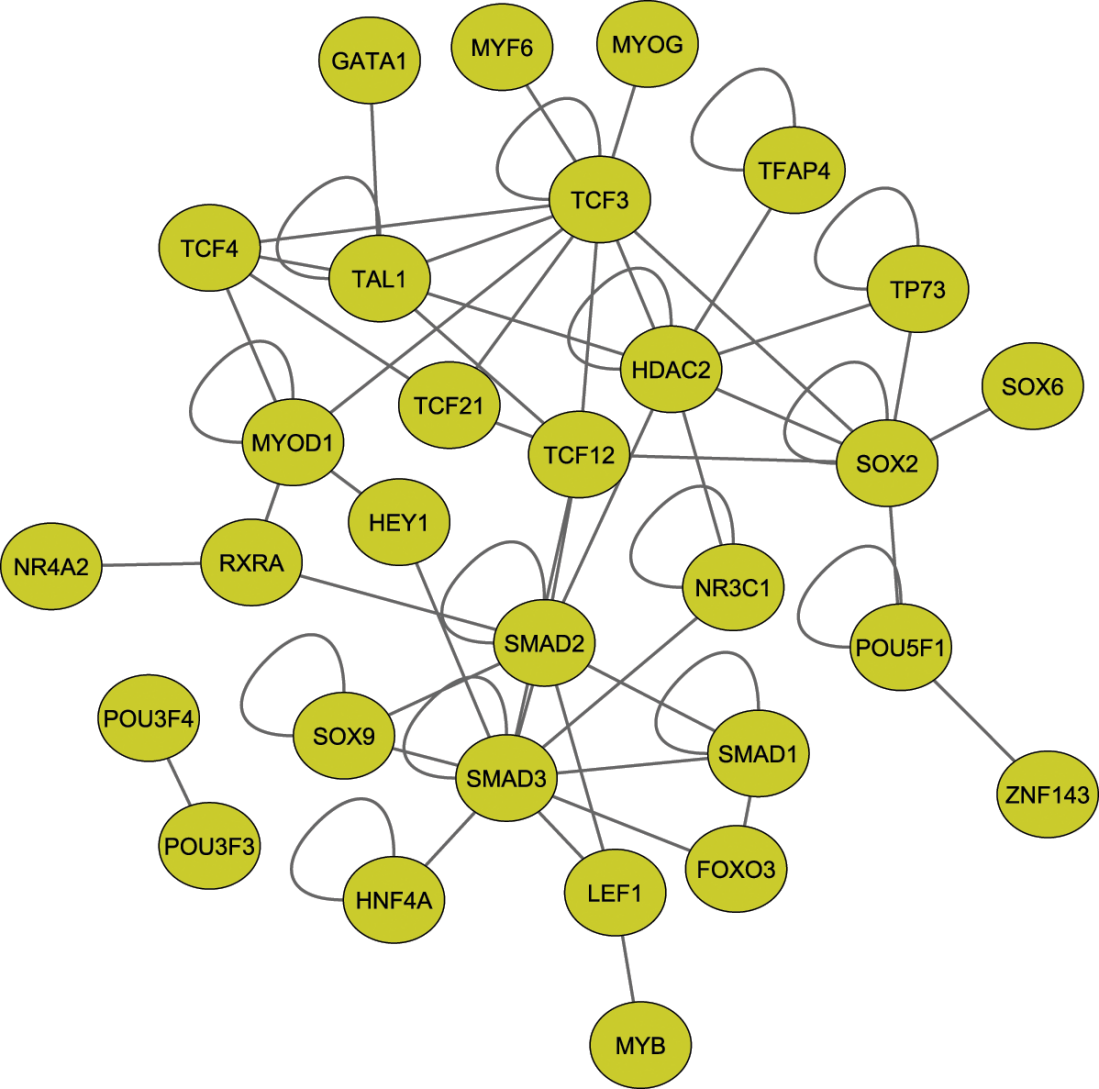
Interaction network of high confidence transcription factors. Protein interaction network between TFs constructed for high confident (p ≤ 0.001, E-value < 10) transcription factors using BioGRID database with TFs belongs to DHS signal overlapped BMo were shown.

Further, in order to prioritize these predicted TFs (S4 Table) and to know potential protein complexes that might be responsible for regulation, we integrated the currently available human protein interaction network from the BioGRID[16] to construct a network of physical associations between TFs predicted to be binding to the *SESN3* gene regulatory regions (see Materials and Methods). This resulted in a network of 67 TFs with 125 associations among them, with TFs like *SMAD3*, *HDAC2*, *TCF3*, *SMAD2*, *CEBPA*, *SOX2*, *SMAD1* and *TAL1* exhibiting high degree of associations (S6 Fig). Such physically interacting TF-TF network could provide potential co-complex interactions contributing to the regulation of *SESN3* gene. While it is possible to argue that Tomtom algorithm we applied for motif comparison, might result in false positives, increasing evidence from large-scale analysis suggests that most of the transcription factors with similar binding sequences tend to regulate genes with similar biological functions[27,51]. It indicates that several of the TFs with very similar binding affinities might be competing to bind to the target sites to result in the final transcriptional outcome. Therefore, in an attempt to identify a high confidence list of TFs, this network was further filtered to include only the TFs which were predicted to bind the BMos with a high confidence (p<e^-03^) from Tomtom analysis and their corresponding motifs overlapping with DHS signals thereby resulting in a subset of TF-TF interactions which are likely to control *SESN3* promoter. The resulting network of 30 nodes with 60 interactions is shown in Fig 4. We found that the hubs of this TF-TF interaction network included *SMAD3*, *TCF3*, *SMAD2*, *HDAC2*, *SOX2*, *TAL1* and *TCF12*. *FOXOs*, which have been documented to regulate the *SESN3* gene transcription[52] were also found to interact with *SMAD3*, suggesting their interplay to combinatorially control *SESN3*.

*SOX2* contains highly conserved DNA binding domains known as HMG (High-mobility group) box domains which facilitate the binding with DNA for transcriptional control [53]. Our study predicted its significant binding (p = 1.75e^-05^, E-value = 0.07) to motif 4. This TF was found physically interacting with *TCF3*, *POU5F1* (*OCT-4*), *SOX6*, *HDAC2* and in addition to that it is also interacting with *TCF12*, thus indirectly bridging with *SMAD3*- another major hub of TF-TF interaction network (Fig 4).

Hepatocytes nuclear factor 4 alpha (*HNF4A*) belongs to the *HNF4* family. It is known to bind to DNA either as a homodimer or as a heterodimer with other transcription factors such as *SMAD3* [54]^,^ [55]. This protein was predicted to be significantly binding to motif 10 (p = 1.66e^-03^, E-Value = 6.67) Protein-protein interaction network data suggests its assistive role in regulating the *SESN3* gene along with *SMADs*.

*SMADs* are signaling cascade associated proteins that act as transcriptional mediators of multiple signaling pathways. For instance–they modulate the transcriptional activity of target genes by transforming growth factor-beta-1[56]. They are reported to bind to CAGA box [57,58] and in some cases to the reverse palindromic sequence ‘GTCTAGAC’ known as SBE (Smad Binding Element) [59] in the upstream regions of target genes by co-complexing with proteins like *SP1*[60], *FOXOs* [54,61], *HNF4A* [55] etc. Motif 4 identified in our analysis was predicted (p = 9.34e^-04^, E-value = 3.76) to be bound by *SMAD3* further supporting the binding specificity of this TF to *SESN3* regulatory regions. *SMAD3* works as a master regulator consistent with our observation that it forms a hub with most other high confident TFs as is evident from our interaction network analysis.

## Conclusions

This work is among the first efforts to identify transcription factor binding sites in the *SESN3* gene promoter using an unbiased computational approach. We found high confidence set of TFs correspond to these identified novel BMos and obtained hubs of TF-TF interaction network that include *SMADs*, *SOXs* and *TCFs*. *FOXOs*, which have been documented to regulate the *SESN3* gene transcription[52] were also found to interact with *SMAD3*, suggesting their interplay to combinatorially control *SESN3*. Some of them including *FOXO3* and *SOX2* have also been experimentally validated for their binding affinity in identified BMos using ChIP-PCR technique. Our findings can form a roadmap to further our understanding on the regulation of the *SESN3* gene.

## Supporting Information

S1 Fig Sestrin 3 is stress response protein, secreted in most of body fluid and liver secretome as shown by mRNA expression pattern of the gene in reference expression data set i.e. mRNA expression profile using genecards.org survey of diverse anatomic regions. (PDF)

S2 Fig Overexpression of *FOXO3* and *SOX2* activated the *SESN3* gene expression in HEK293 cells. (PDF)

S3 Fig Identification of potential binding motifs by phylogenetic footprinting of 3 kb upstream and 2 kb instream regulatory regions of *SESN3* gene. (PDF)

S4 Fig DNase I hypersensitive sites and block diagram showing the occurrence of conserved motifs in human and mouse. DNase I hypersensitive region was shown in 3 kb upstream and 2 kb instream sequences of *SESN3* in (A) human cell lines and (B) mouse liver (8 week adult and 14.5 days embryo) using ENCODE project, represented by UCSC browser visualization tool. An overlap of DHS signal was found and shown as dark band over respective motifs in block diagram. The combined best matches of a sequence to a group of motifs were shown by combined p value. Sequence strand specified as “+” (input sequence was read from left to right) and “-” (input sequence was read on its complementary strand from right to left) with respect to the occurrence of motifs. The two coordinates on x-axis represents the 3 kb upstream and 2 kb instream regions as base distance (in blue) and genic distance (with respect to gene start site, in red) of *SESN3* gene.
(PDF)

S5 Fig Length distribution of TF sites overlapping with the width of discovered motifs. (PDF)

S6 Fig Interaction network of predicted transcription factors. Protein interaction network between TFs constructed for all possible predicted transcription factors using BioGRID database with TFs belongs to DHS signaled BMo were shown in asterisk “*”.
(PDF)

S1 Table This table enlists human-*SESN3* in orthologous species (primates and rodents) with its location (coordinates) and % query, target matching. (XLSX)

S2 Table Motifs identified in 5kb upstream region of *SESN3* in occuring species was shown. For each motif, significance, location from start (0 in block diagram) and sequence at location for each species was documented.
(XLSX)

S3 Table Correlation indices of motifs identified in analysis for 5 kb upstream (20 motifs) were shown. (XLSX)

S4 Table This table enlists TFs associated to 20 motifs identified in 5kb upstreams region of *SESN3* predicted by Tomtom analysis using Jolma et al, Jaspar & UniPROBE Mouse_2014 and TRANSFAC databases (Motifs supported by DHS signal were color coded; green in mouse and yellow in human). (XLSX)

S5 Table This table enlists TFs associated to DHS supported motifs identified in 3kb upstream and 2 kb instreams regulatory region of SESN3 predicted by Tomtom analysis using Jolma et al, Jaspar & UniPROBE Mouse_2014 and TRANSFAC databases (Motifs supported by DHS signal were color coded; green in mouse, yellow in human and peach in both). (XLSX)
