# Supplementary figures and images for: Prediction and Validation of Transcription Factors Modulating the Expression of Sestrin3 Gene Using an Integrated Computational and Experimental Approach
Source: PLoS One. 2016 Jul 28;11(7):e0160228. doi: 10.1371/journal.pone.0160228 (PMC4965051; doi:10.1371/journal.pone.0160228)

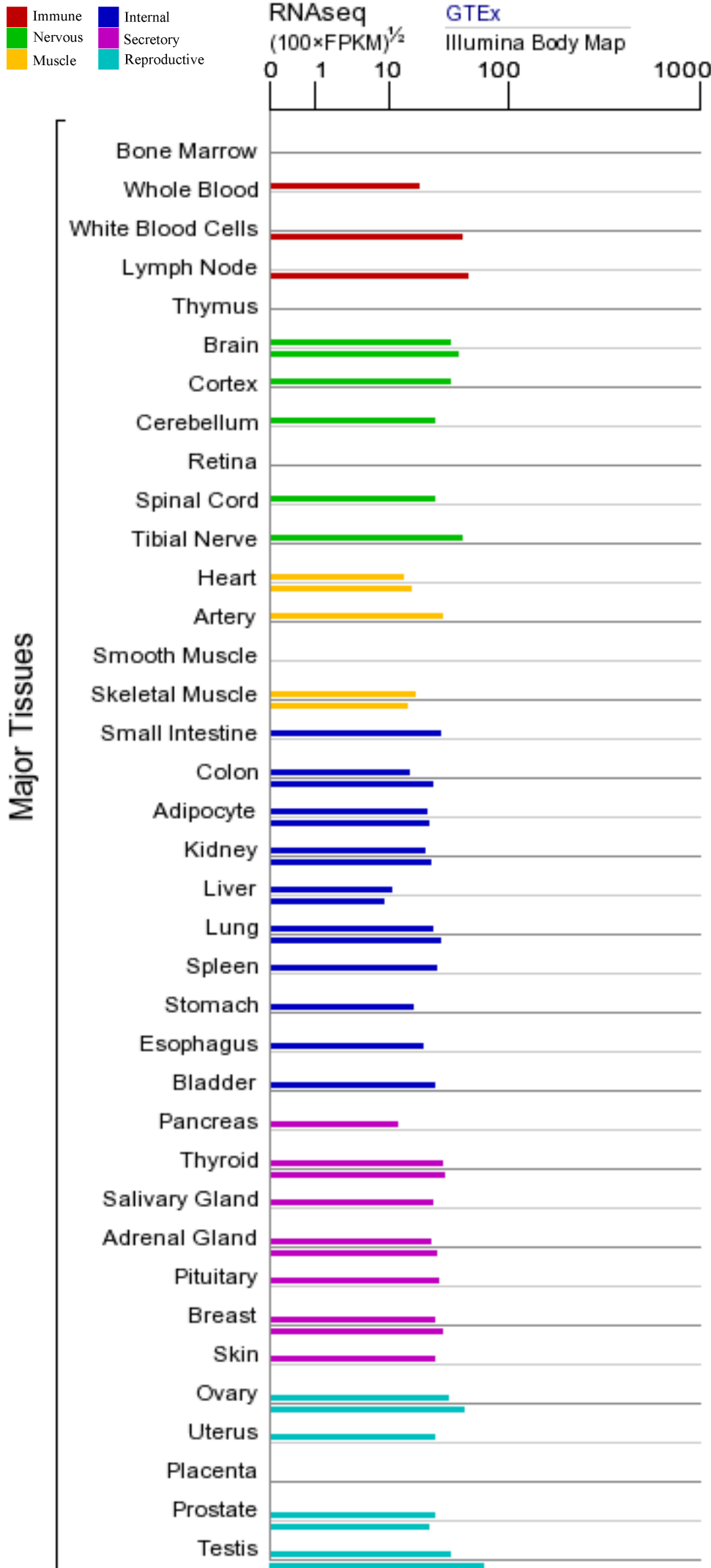

Supplement: S1 Fig — (PDF) [file pone.0160228.s001.pdf]

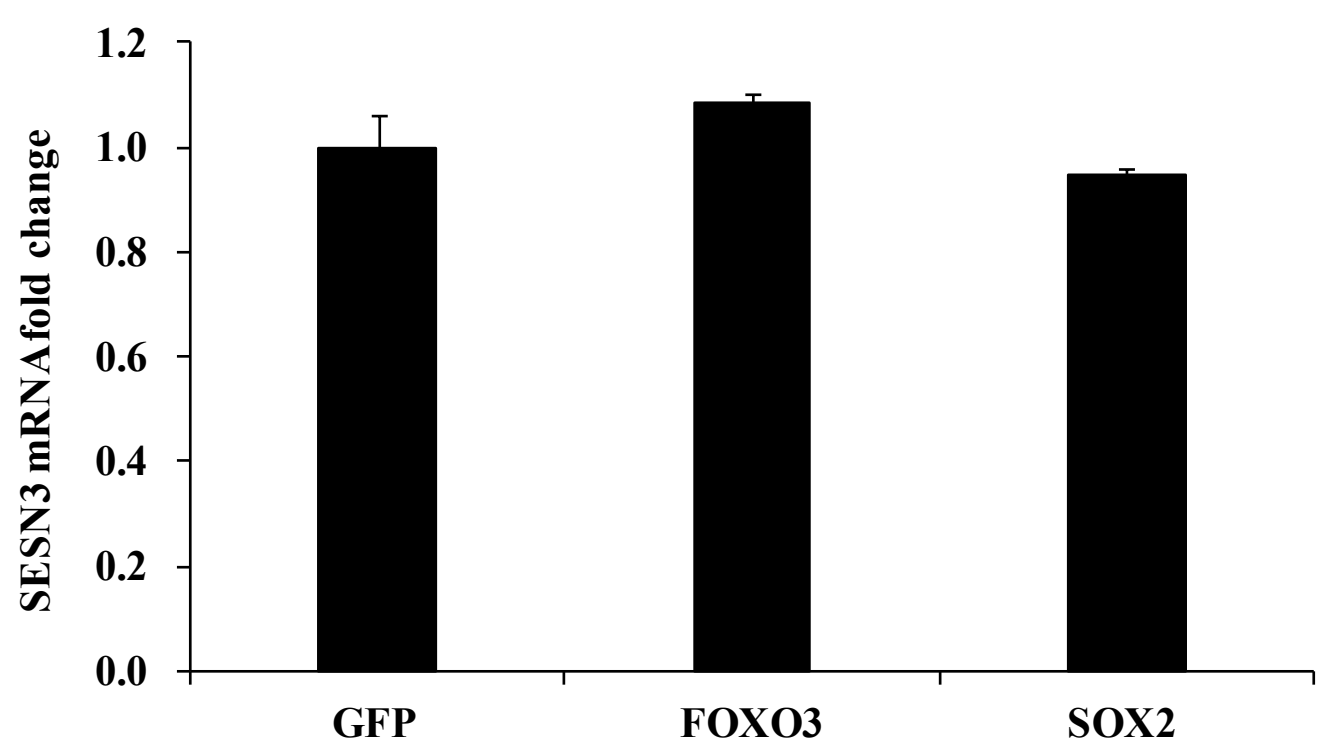

Supplement: S2 Fig — (PDF) [file pone.0160228.s002.pdf]

# Distribution of overlapping motifs

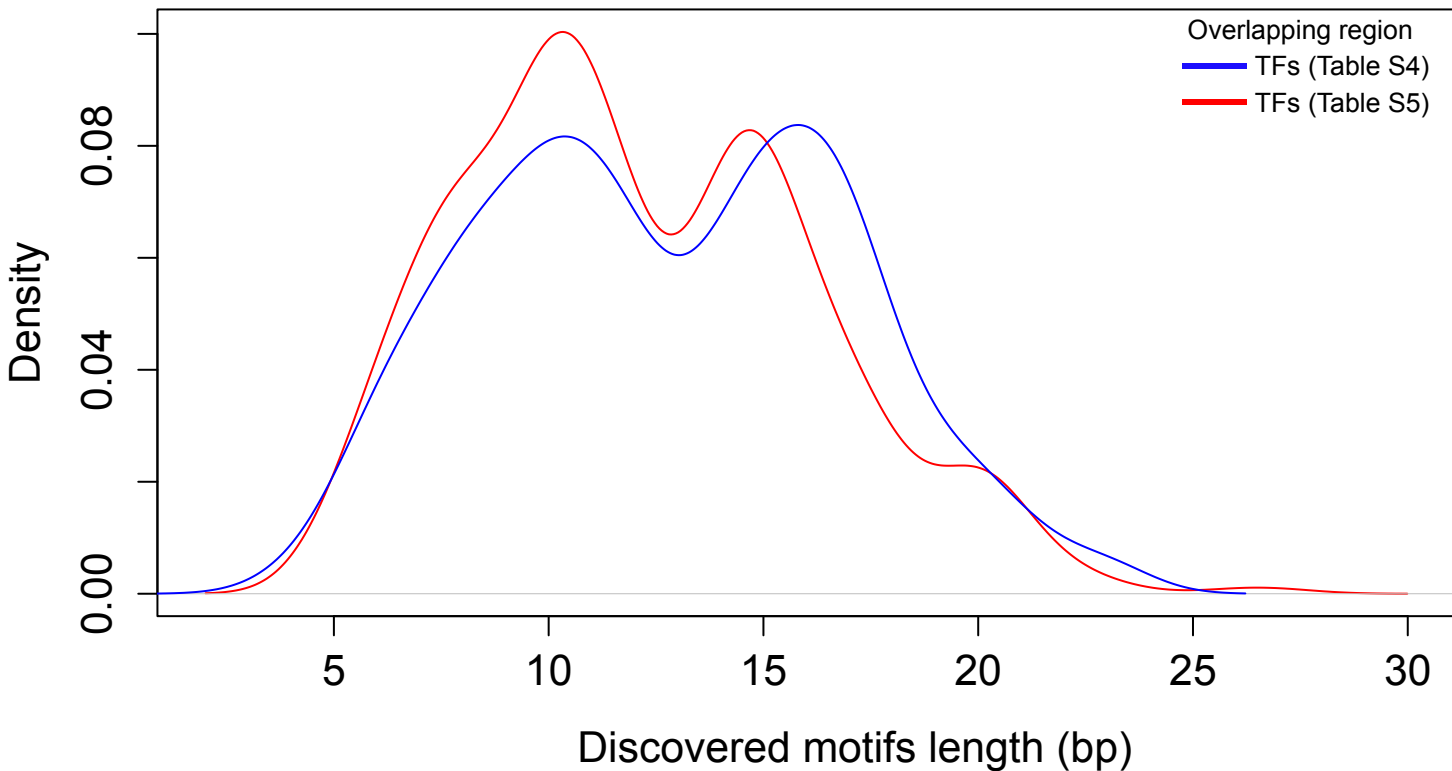

Supplement: S5 Fig — (PDF) [file pone.0160228.s005.pdf]

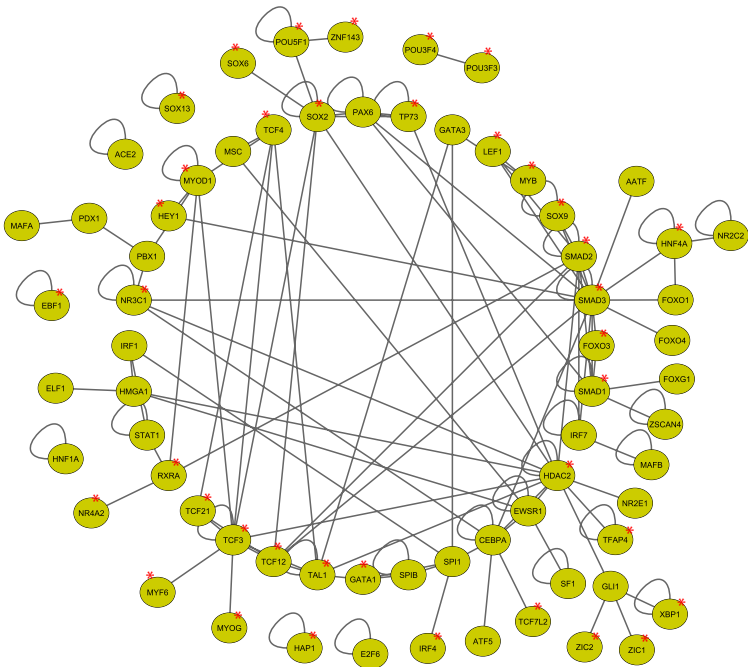

Supplement: S6 Fig — Protein interaction network between TFs constructed for all possible predicted transcription factors using BioGRID database with TFs belongs to DHS signaled BMo were shown in asterisk “*”. (PDF) [file pone.0160228.s006.pdf]
