# Supplementary material for: Prediction and Validation of Transcription Factors Modulating the Expression of Sestrin3 Gene Using an Integrated Computational and Experimental Approach
Source: PLoS One. 2016 Jul 28;11(7):e0160228. doi: 10.1371/journal.pone.0160228 (PMC4965051; doi:10.1371/journal.pone.0160228)

Motif 1
5.4E-74
9 sites

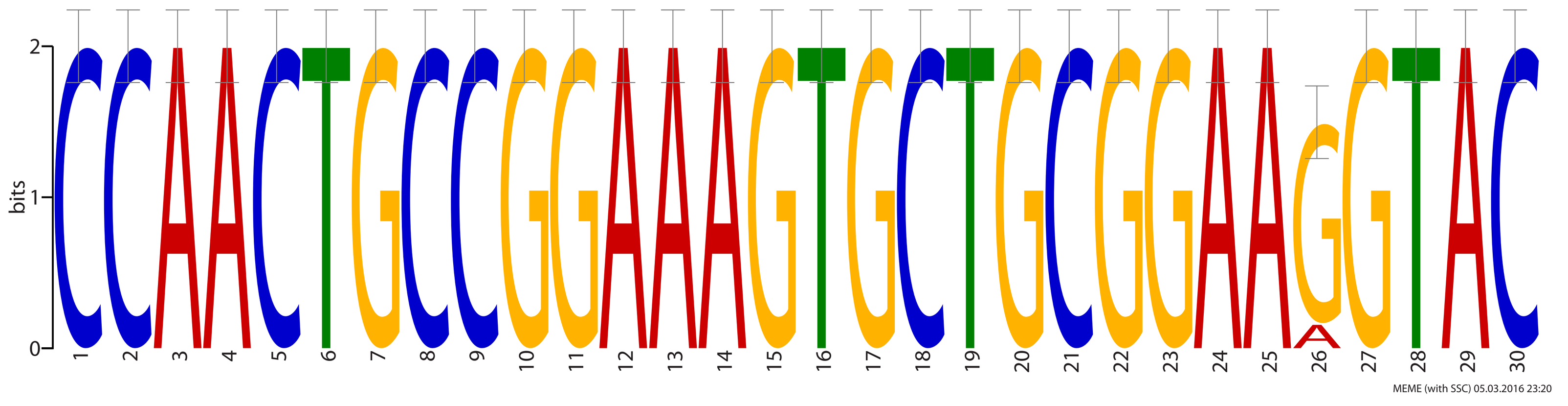

Motif 2
1.1E-72
9 sites

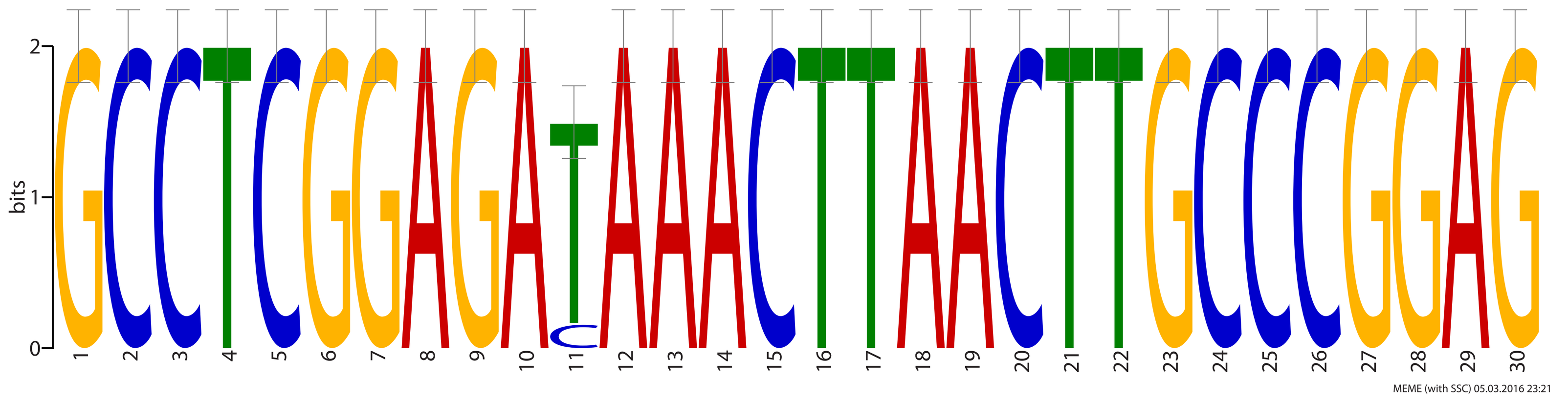

Motif 3
1.9E-59
9 sites

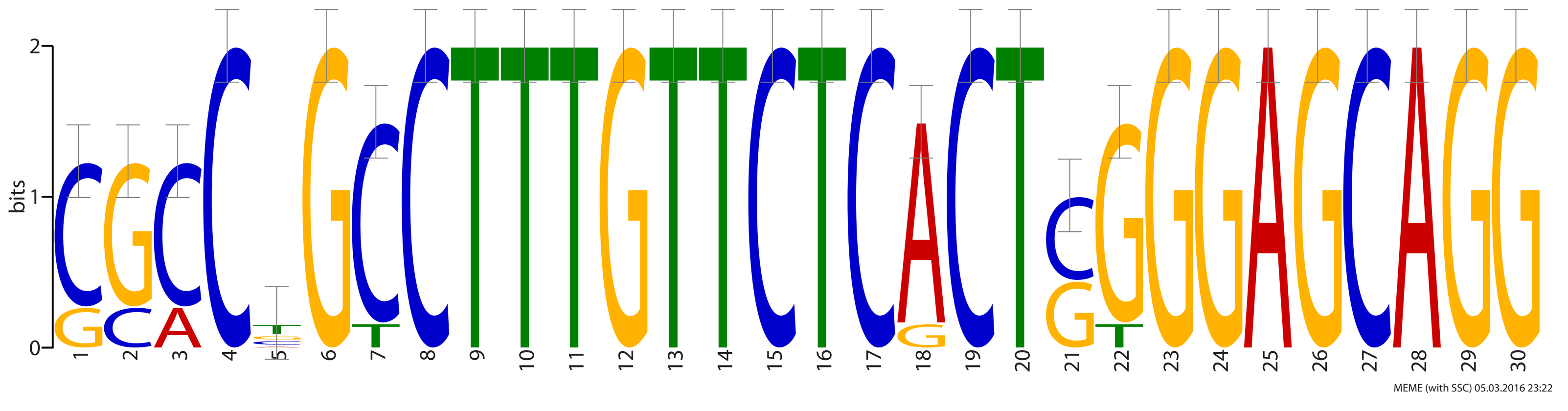

Motif 4
2.5E-58
9 sites

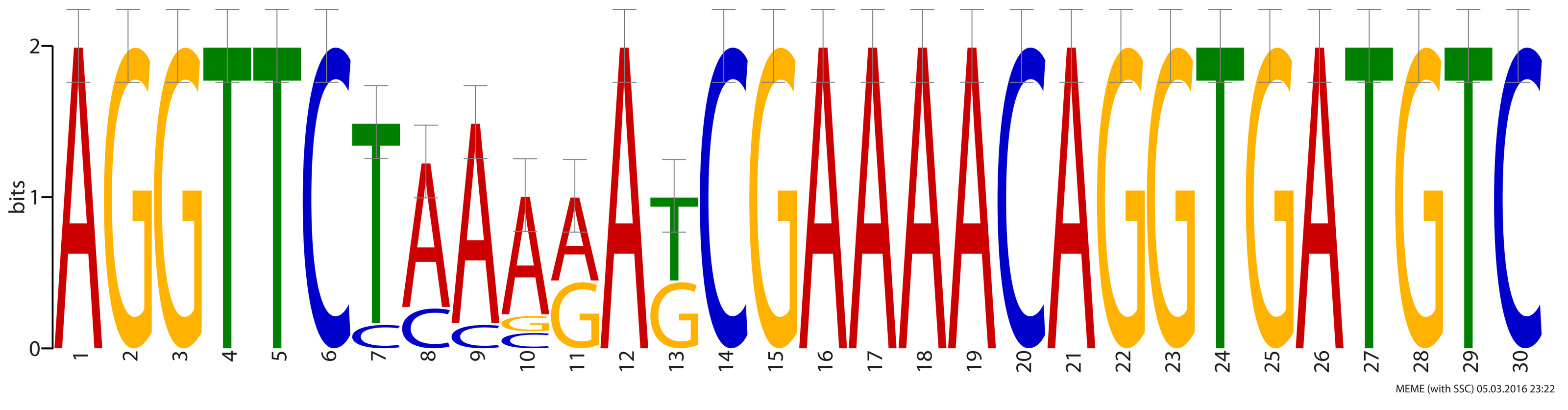

Motif 5
4.7E-55
9 sites

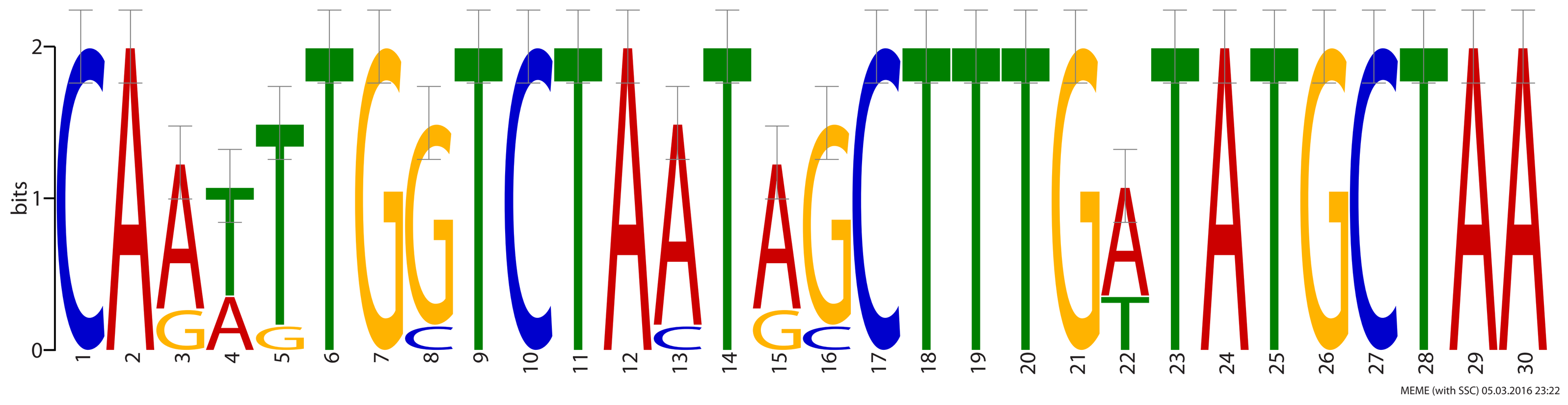

Motif 6
8.4E-53
9 sites

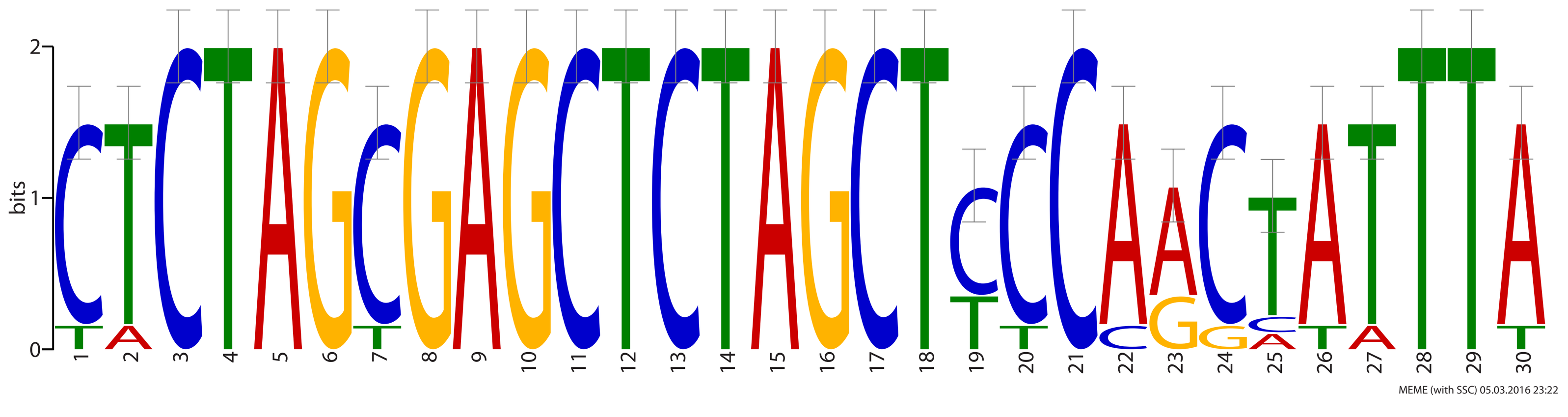

Motif 7
1.5E-49
11 sites

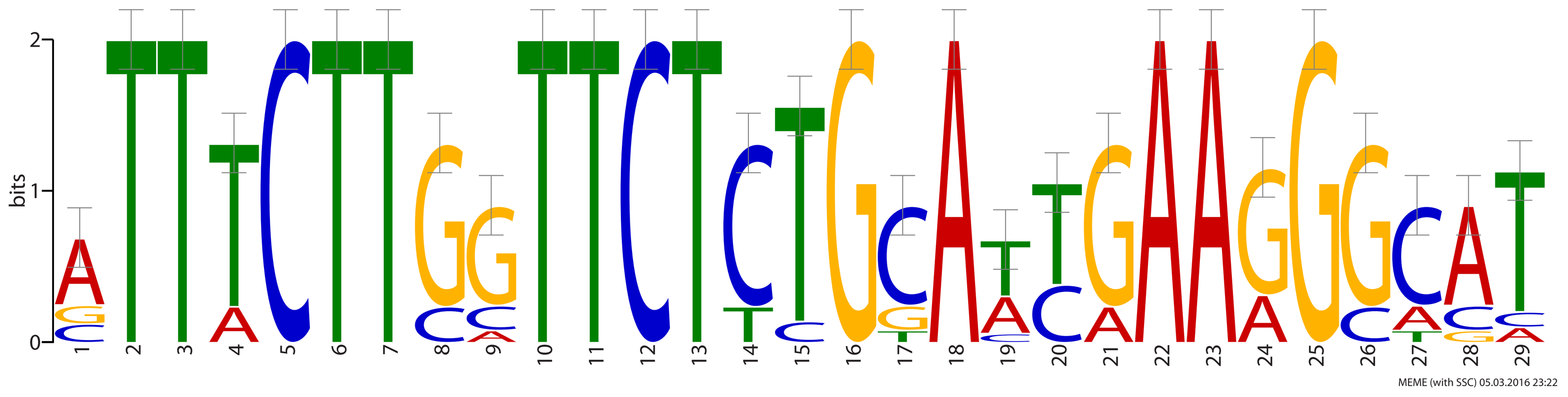

Motif 8
2.6E-51
9 sites

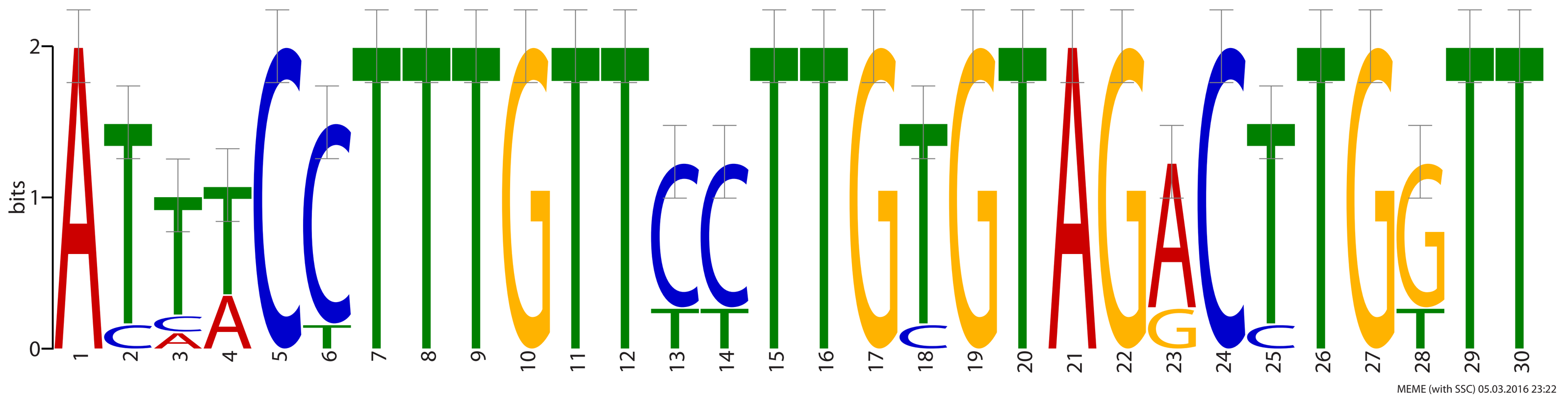

Motif 9
5.4E-53
9 sites

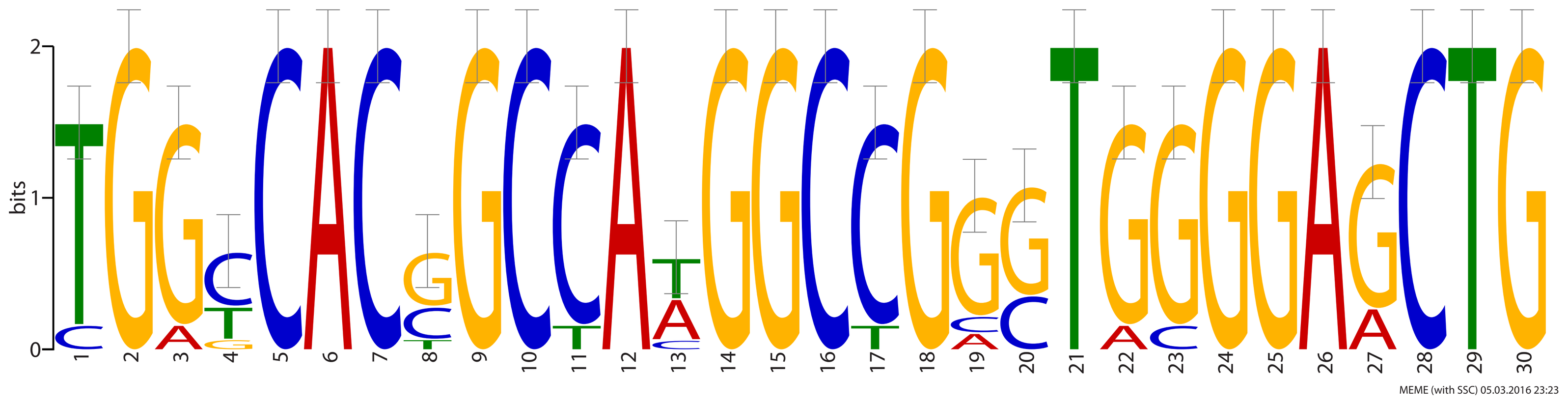

Motif 10
4.4E-50
9 sites

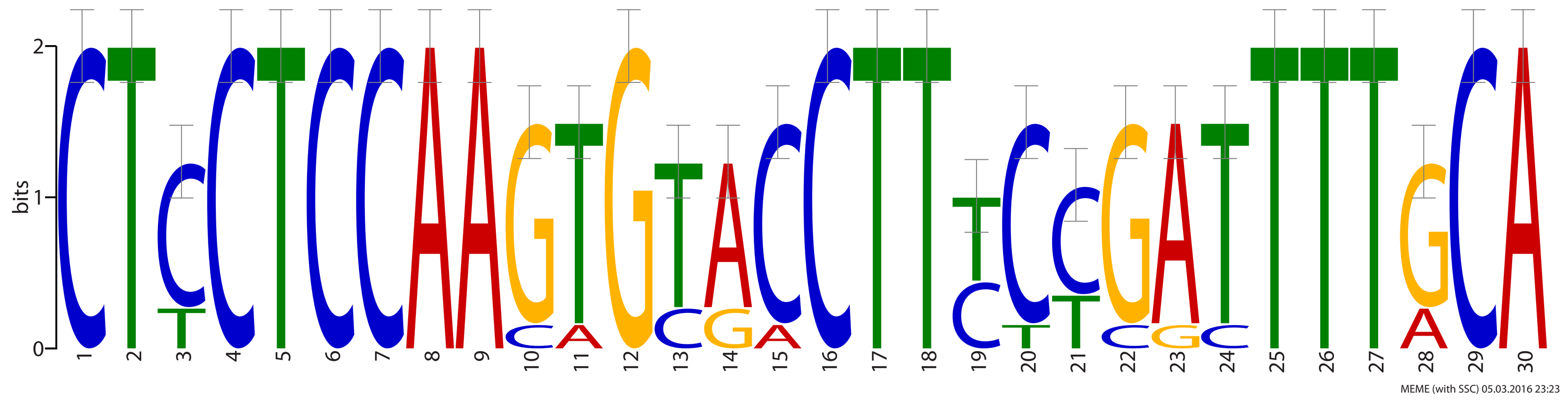

Motif 11
1.7E-52
9 sites

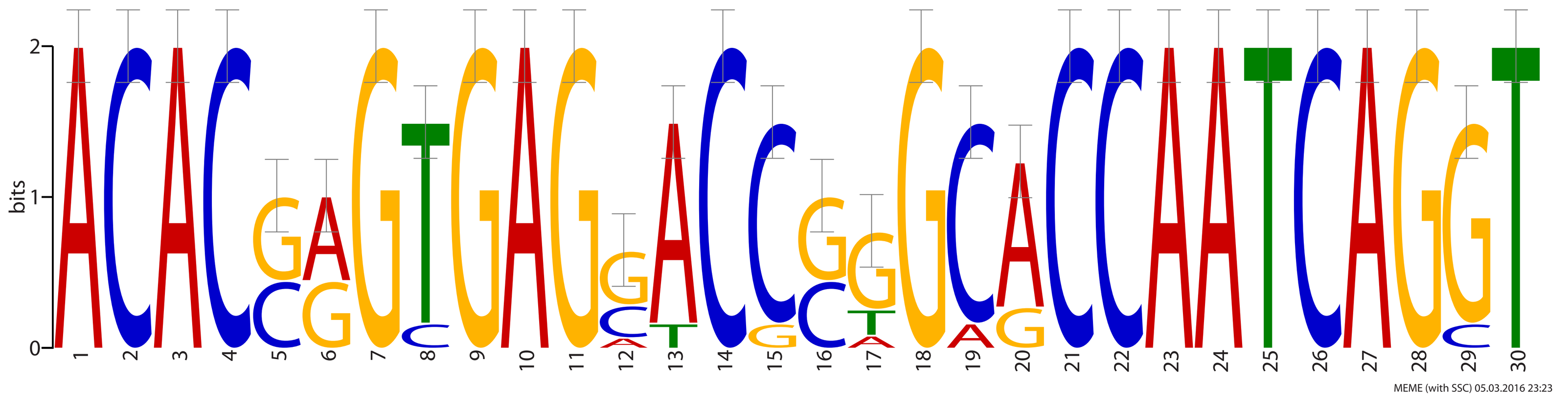

Motif 12
1.8E-47
8 sites

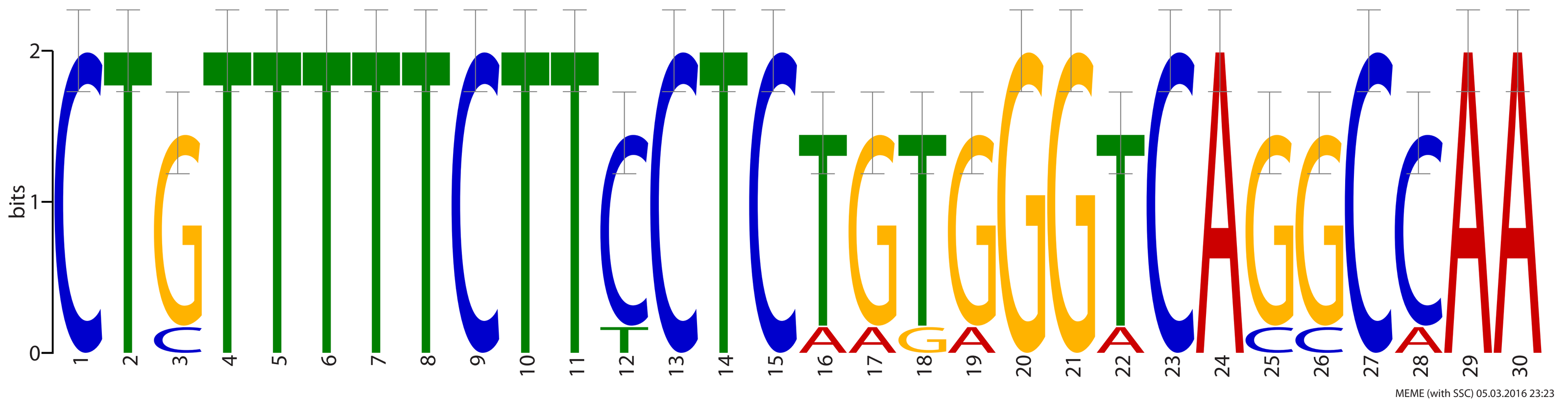

Motif 13
2.5E-51
9 sites

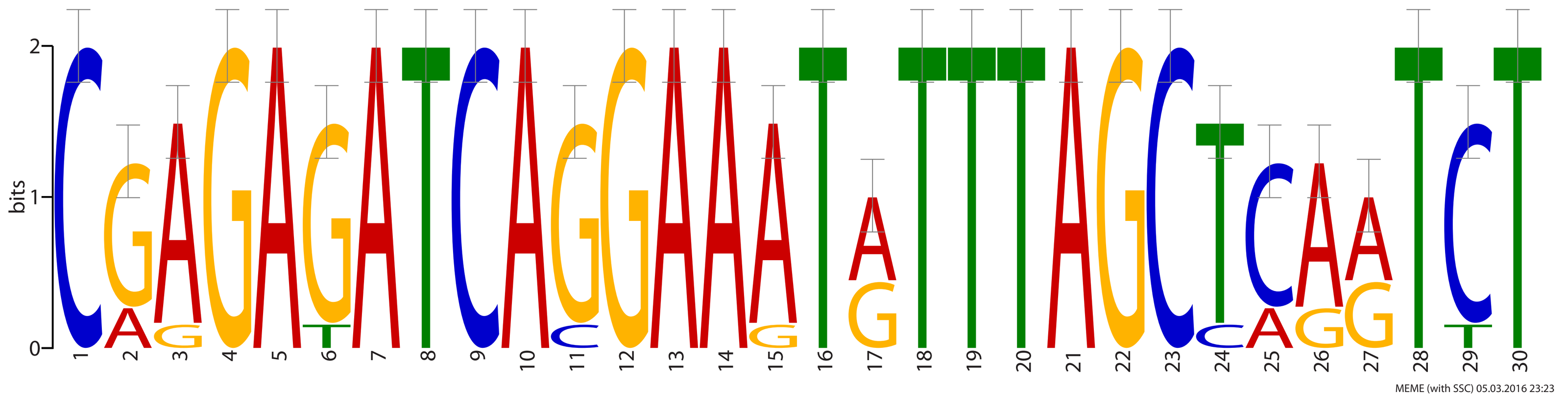

Motif 14
2E-47
10 sites

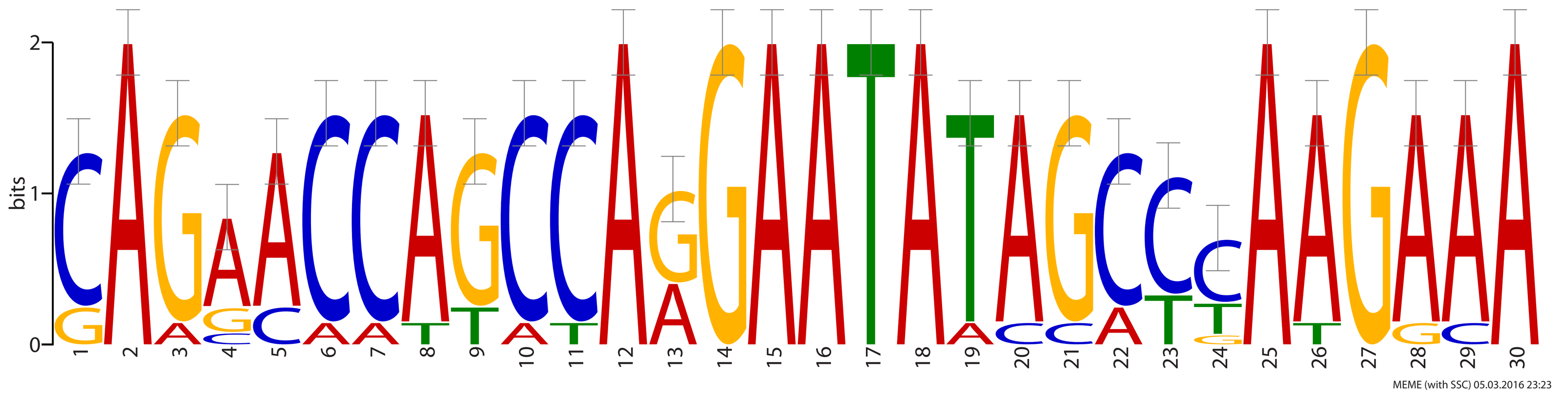

Motif 15
4.2E-45
9 sites

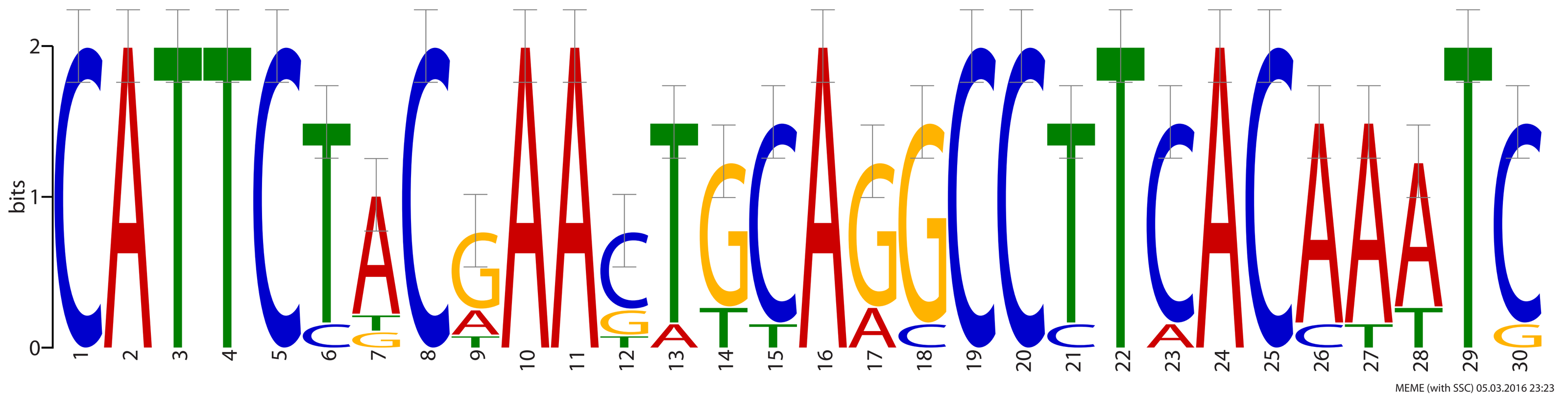

Motif 16
9.1E-45
8 sites

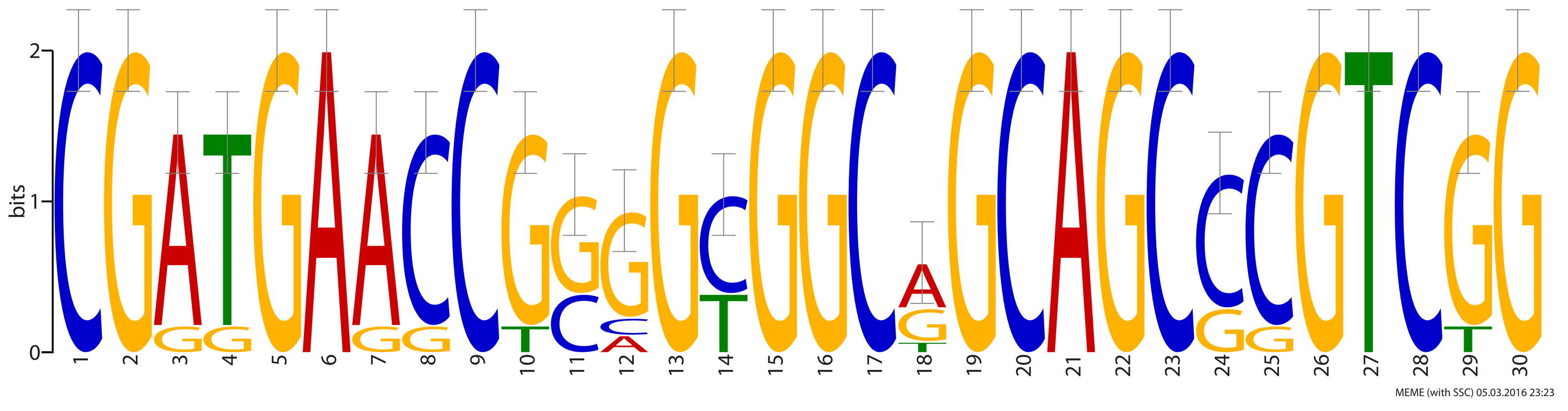

Motif 17
1.8E-44
8 sites

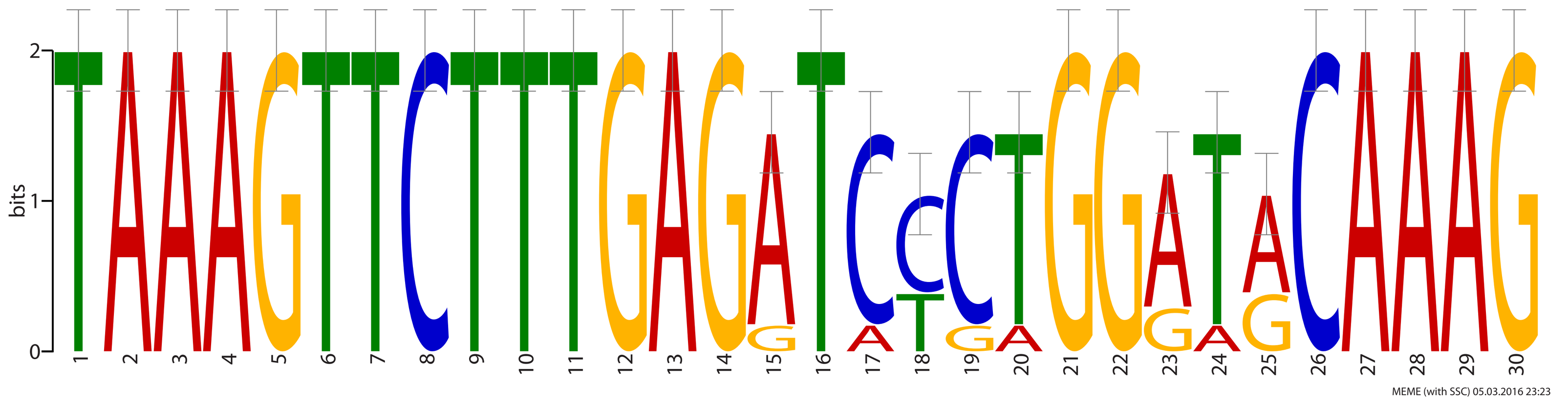

Motif 18
8.9E-45
7 sites

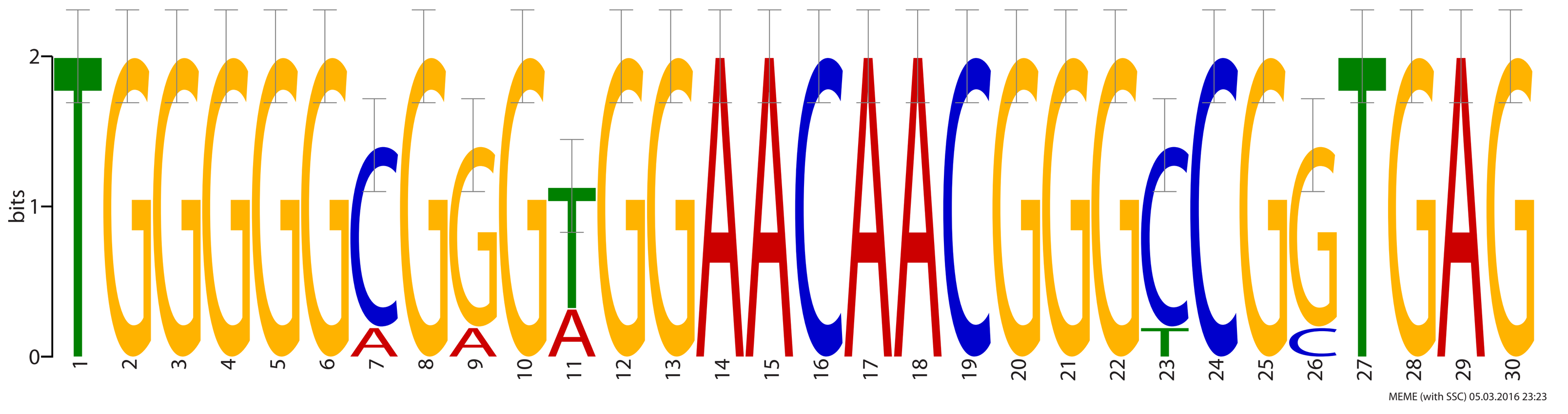

Motif 19
1.6E-44
7 sites

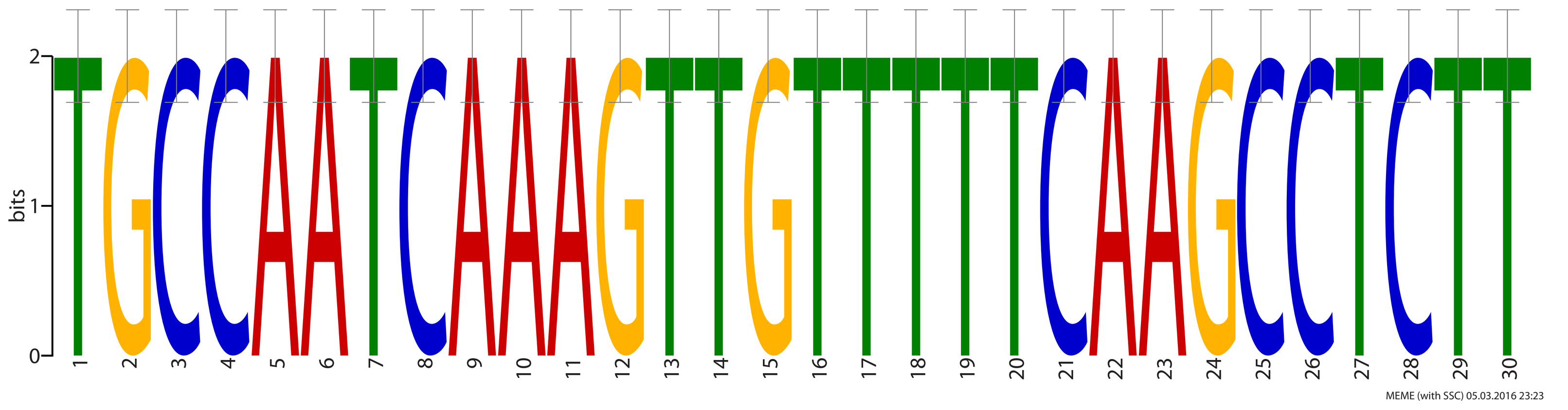

Motif 20
9.2E-44
8 sites

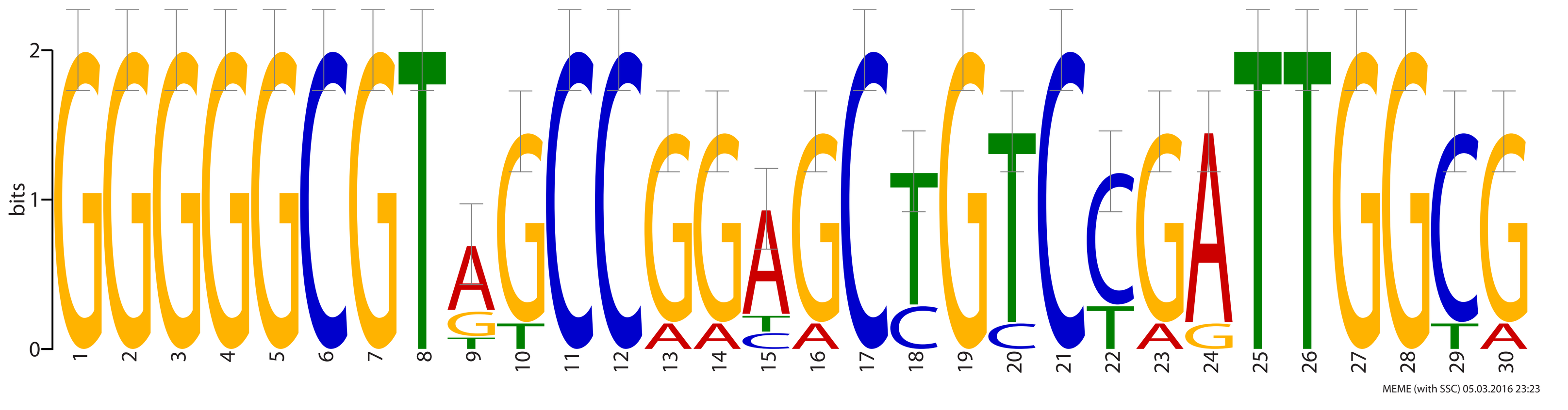

Supplement: S3 Fig — (PDF) [file pone.0160228.s003.pdf]
