# Supplementary material for: Prediction and Validation of Transcription Factors Modulating the Expression of Sestrin3 Gene Using an Integrated Computational and Experimental Approach
Source: PLoS One. 2016 Jul 28;11(7):e0160228. doi: 10.1371/journal.pone.0160228 (PMC4965051; doi:10.1371/journal.pone.0160228)
Supplement: S4 Fig — DNase I hypersensitive region was shown in 3 kb upstream and 2 kb instream sequences of SESN3 in (A) human cell lines and (B) mouse liver (8 week adult and 14.5 days embryo) using ENCODE project, represented by UCSC browser visualization tool. An overlap of DHS signal was found and shown as dark band over respective motifs in block diagram. The combined best matches of a sequence to a group of motifs were shown by combined p value. Sequence strand specified as “+” (input sequence was read from left to right) and “-” (input sequence was read on its complementary strand from right to left) with respect to the occurrence of motifs. The two coordinates on x-axis represents the 3 kb upstream and 2 kb instream regions as base distance (in blue) and genic distance (with respect to gene start site, in red) of SESN3 gene. (PDF) [file pone.0160228.s004.pdf]

A

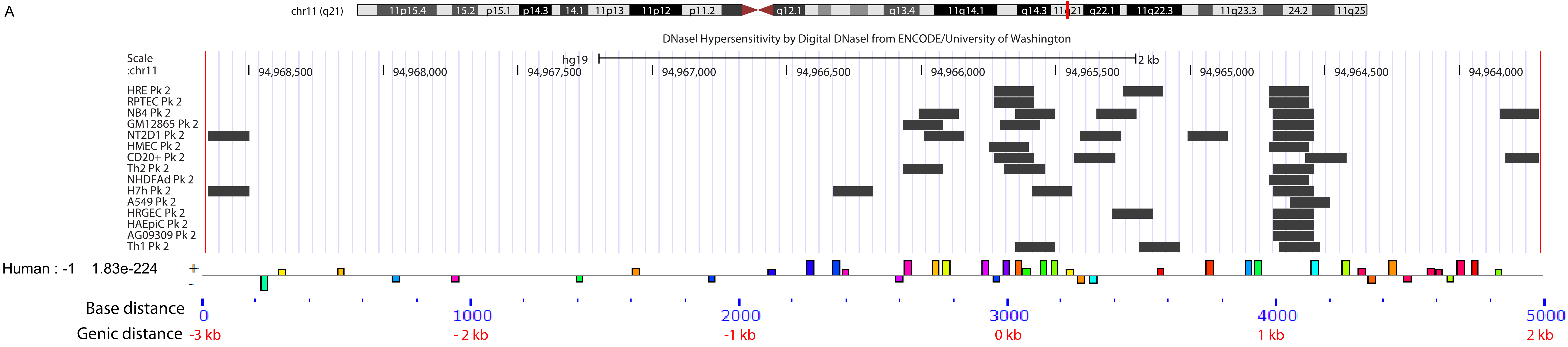

B

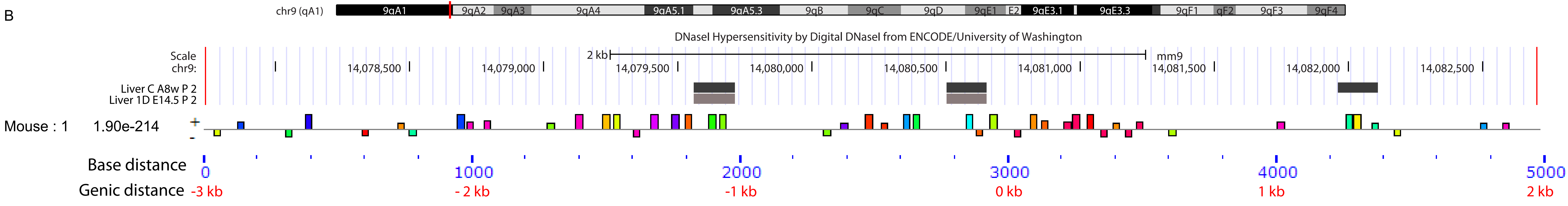

Motif 1 Motif 2 Motif 3 Motif 4 Motif 5 Motif 6 Motif 7 Motif 8 Motif 9 Motif 10 Motif 11 Motif 12 Motif 13 Motif 14 Motif 15 Motif 16 Motif 17 Motif 18 Motif 19 Motif 20
